# The Social Environment Matters for Telomere Length and Internalizing Problems During Adolescence

**DOI:** 10.1007/s10964-023-01848-w

**Published:** 2023-09-25

**Authors:** Darlene A. Kertes, Cherita Clendinen, Ke Duan, Jill A. Rabinowitz, Christopher Browning, Peter Kvam

**Affiliations:** 1 Department of Psychology, University of Florida, 945 Center Drive, Gainesville, FL 32611-2250 USA; 2 Genetics Institute, University of Florida, 945 Center Drive, Gainesville, FL 32611-2250 USA; 3 Department of Mental Health, Johns Hopkins Bloomberg School of Public Health, 624 N. Broadway, Baltimore, MD 21205 USA; 4 Department of Sociology, Ohio State University, 1885 Neil Ave, Columbus, OH 43210 USA

**Keywords:** Telomere length, Depression, Anxiety, Stress, Family support, School belongingness

## Abstract

Depression and anxiety symptoms are on the rise among adolescents. With increasing evidence that cellular aging may be associated with depressive and anxiety symptoms, there is an urgent need to identify the social environment context that may moderate this link. This study addresses this research gap by investigating the moderating role of the social environment on the relation between telomere length and emotional health among adolescents. Participants were 411 non-Hispanic (88.56%) Black (100%) adolescents (*M* = 14.23 years, SD = 1.85, female = 54%) in a major metropolitan city. Youth and parents reported on an array of social risk and protective factors, and youth provided DNA samples for telomere length measurement. Results demonstrated that the association of telomere length and anxiety symptoms was stronger among youth with higher perceived stress or lower school belongingness, and the association of telomere length with depressive symptoms was stronger under conditions of higher parent inter-partner psychological aggression. The results enhance our understanding of the complex associations between biological aging, the social environment, and mental health in adolescence.

## Introduction

Over the past decade, there has been a dramatic rise in the number of Black children and adolescents who report symptoms of anxiety and depression (SAMHSA, 2019), and these youth often manifest more severe and chronic symptoms than their peers (BCBS & BHI, 2022). Despite the rising burden on Black youth of depressive and anxiety symptoms (collectively known as internalizing problems), there remains a gap in knowledge regarding the individual-specific and contextual factors that may attenuate or exacerbate these symptoms. This study examined individual differences in telomere length, a biomarker of cellular aging, along with an array of contextual factors in the social environment to shed new light on the complex interplay of factors implicated in internalizing problems among Black youth, which may inform prevention and intervention strategies in this population. Stated differently, the present study tested whether risk and protective factors in the social environment matter for coupling of stress-linked health outcomes at the biological and behavioral level.

### Telomere Length Associations with Internalizing Symptoms

Telomeres are nucleoprotein complexes of DNA sequences found at the end of chromosomes that act to protect DNA from damage during replication (McEachern et al., 2000). Although it is normal for telomeres to shorten during replication as part of chronological aging, exposure to chronic stressors can lead to accelerated telomere shortening via exposure to oxidative stress and subsequent cellular damage (von Zglinicki, 2002). Telomere erosion occurs most rapidly in the first two decades of life compared to any other point in the lifespan (Factor-Litvak et al., 2016). Extant literature reviews (Coimbra et al., 2020; Colich et al., 2020) and individual studies show early adverse life experiences predict telomere length in childhood (e.g., Beijers et al., 2020; Ridout et al., 2019) and adolescence (Chen et al., 2019; Tung et al., 2022), with findings largely showing that greater exposure to childhood adversity is associated with shorter telomere length. Stated conceptually, chronic stress can speed up the process by which the protective ends of chromosomes shorten, leading to faster-aging tissues and increased risk for diseases and disorders. In this way, telomere length can be viewed as a biological reflection of the accumulation of life experiences, including early life stressors and adversities known to increase risk for mental health symptoms such as internalizing problems (DelGiudice, 2018).

Telomere length has previously been associated with internalizing symptoms (Verhoeven et al., 2018; Wade et al., 2020), among other myriad adverse health outcomes (i.e., morbidity and mortality; Blasco, 2005; Schneider et al., 2022). Multiple clinical and community-based samples indicate that individuals with greater anxiety and depressive symptoms and disorders evidenced shorter telomere length relative to unaffected individuals (e.g., Ford et al., 2023; Malouff & Schutte, 2017) although across the literature, findings remain mixed with null associations also reported (Cerveira de Baumont et al., 2021; Needham et al., 2015). Discrepancies in study findings may be because of sample characteristics such as age, gender, and race/ethnicity (Coimbra et al., 2020; Zhu et al., 2011) in addition to a variety of contextual factors that may influence these associations (Burgin et al., 2019).

### Environmental Characteristics as Potential Moderators

Whereas shorter telomere length has been linked to internalizing symptoms, not all youth who evidence shorter telomere length have internalizing problems, underscoring the importance of the social environment in influencing these associations. Consistent with both the social determinants of health framework and developmental ecological systems theory, it is widely believed that multi-level environmental influences shape developmental outcomes (Alegria et al., 2018). Among social factors, perceived discrimination (Liu et al., 2017; Sharma et al., 2019), poor relationships with family or peers (Gaylord-Harden et al., 2006; Gray et al., 2010), or greater exposure to neighborhood violence (Kliewer et al., 2004) have been tied to increased risk for internalizing symptoms among Black adolescents.

To date, no studies examine how the social context might influence telomere length—internalizing symptoms, particularly among marginalized Black youth during adolescence. Adolescence is characterized by increased plasticity, heightened reactivity and sensitivity to social interactions, profound changes in neurobiology (Hankin, 2015; Holder & Blaustein, 2014), as well as exposure to a range of stressors which may act as moderators of the associations between telomere length and internalizing problems. Consistent with the weathering hypothesis, repeated exposures to and accumulation of stressors may lead to cellular damage and accelerated telomere erosion (Geronimus et al., 2006), which may exacerbate risk for psychological impairments. Given that Black individuals are disproportionately burdened by unaddressed internalizing problems (BCBS & BHI, 2022; Cummings & Druss, 2011) and aging diseases linked to telomere length in adulthood (Shammas, 2011; Xu et al., 2020), an examination of malleable contextual factors that may influence the association of telomere length and internalizing symptoms could be targeted to inform prevention and intervention efforts and reduce health disparities.

## Current Study

The current study sought to address a number of gaps in the literature. First, although numerous studies have examined social environment predictors of telomere length and internalizing symptoms individually, little is known about risk and protective contextual factors that might exacerbate or buffer the association between telomere length and internalizing problems. Second, these associations are examined during adolescence, a developmental stage characterized by both marked changes in the social environment and increased vulnerability to internalizing problems. Third, because minority youth in the United States are disproportionately impacted by stressors linked with socioeconomic disadvantage, and because risk and protective factors may differ for minority youth, this study examined the relations between social context, telomere length, and internalizing symptoms in a sample of Black youth. It was hypothesized that telomere length, as a biomarker of cellular aging reflecting cumulative effects of prior life experience, would interact with social environment factors assessed in adolescence. As the study was cross-sectional in nature, higher TL—internalizing associations may reflect a stronger coupling of dysregulation at biological and behavioral levels. This study capitalized on a Bayesian regression approach, which allows for assigning a probability to each predictor quantifying how credibly it is related to the outcome. This approach yields conceptually simpler conclusions compared to regression-based modeling (Kruschke, 2014), quantifies the relative certainty/ uncertainty about the relations among variables, and enables evaluation for evidence for the null (i.e., the absence of a relationship; Wagenmakers, 2007), yielding more coherent conclusions than traditional null hypothesis testing. Given the novelty of examining social moderators of telomere length with internalizing problems in this study, in lieu of specific hypotheses about interaction effects, Bayesian analysis was utilized to determine predictors with highest confidence given the totality of the data.

## Methods

### Participants

Participants in this study were youth between the ages of 11–17 along with their primary caregiver. Participants were recruited using school- and vendor-based address lists from a contiguous region of a large metropolitan area in Columbus, Ohio. For participants with more than one child in the target age range, one child was randomly selected to participate. Institutional Review Boards at the Ohio State University and the University of Florida approved the study. A total of 437 Black youth provided data for this study (phenotype and salivary samples). Of those, sufficient quantity and quality of DNA was obtained from 417, of which 411 were successfully quantified for telomere length, reflecting the final sample size in this report.

### General Procedure

Data collection for the study occurred in participants’ homes. Following written parental consent and youth assent, trained interviewers conducted surveys with youth and caregivers separately. Youth- and parent-report measures were administered via a combination of interviewer-assisted and self-administered surveys. Saliva samples were obtained via passive drool using DNA Genotek OGD-500 collection device (Ottawa, Canada). Samples were held at the survey research center at −20 °C and stored on dry ice to −80 °C freezers at the Ohio State University and transferred to the University of Florida where they were maintained at −80 °C until assay.

### Measures

#### Demographics

Youth participants reported their age, sex, and race. Caregivers reported family demographics, including caregiver age and education, annual household income, and marital status.

#### Anxiety symptoms

Anxiety symptoms were measured using the anxiety items from the Patient-Reported Outcomes Measurement Information System (PROMIS short form; Pilknois et al., 2011). Participants were asked to report their experiences of emotional distress during the past week on a 5-point Likert scale (1 = Never, 2 = Rarely, 3 = Sometimes, 4 = Often, 5 = Always). Participants responded to statements that assessed current anxiety symptoms (e.g., “I felt fearful”, “My worries overwhelmed me”, “I felt like I needed help for my anxiety”). The scale consisted of 8 items (*M* = 1.76, SD = 0.71) which had good internal consistency (Cronbach’s *α* = 0.86).

#### Depressive symptoms

Depressive symptoms were measured using the Center for Epidemiological Studies Depression Scale Short Form (CES-D Short Form; Cole et al., 2004). Participants were asked to use a 4-point Likert scale (0 = Rarely, 1 = Some of the time, 2 = Occasionally, 3 = Most or all the time) to indicate the frequency of affective or depressed moods experienced during the past week. Participants responded to statements that assessed current depressive symptoms (e.g., “I felt lonely”, “I thought my life had been a failure”, “I felt that everything I did was an effort”). The scale consisted of 9 items (*M* = 0.82, SD = 0.56), which had good internal consistency (Cronbach’s *α* = 0.73).

#### Social environment moderators

An array of social environment features was included as potential moderators of the association of telomere length with anxiety and depressive symptoms. These include personal, family, interpersonal, and neighborhood risk and protective factors. Table 1 indicates constructs included in the analyses, including the scale origin, number of items comprising each measure, and internal consistency. Internal consistency criteria for inclusion in analyses were Cronbach’s *α* > 0.6 for scales with 3+ items and inter-item correlations *r* > 0.2 for 2-item scales (Sijtsma, 2009).

**Table 1:** Measures of social environment risk and protective factors

Variable (Reporter)	Scale (No. items)	Citation	Cronbach’s *α* / *r*
Individual			
Self-control (Y)	MRSS (9)	Wikström and Svensson (2010)	0.70
Attitude of violence (Y)	COV (7)	Stewart and Simons (2006)	0.78
Loneliness (Y)	LQ-SS (9)	Asher and Wheeler (1985)	0.91
Personal religiosity (Y)	ADD Health (2)	Harris et al. (2019)	*r* = 0.50
Religious activity participation (Y)	ADD Health (2)	Harris et al. (2019)	*r* = 0.54
Shame if caught by teachers (Y)	MRSS (5)	Wikström and Svensson (2010)	0.85
Shame if caught by parents (Y)	MRSS (5)	Wikström and Svensson (2010)	0.88
Shame if caught by friends (Y)	MRSS (5)	Wikström and Svensson (2010)	0.91
Family			
Family environment (CG)	FES (9)	Moos and Moos (1994)	0.62
Family support (Y)	SSS (6)	Turner et al. (1983)	0.78
Family care (Y)	ADD Health (3)	Harris et al. (2019)	0.90
Depression (CG)	CES-D (9)	Cole et al. (2004)	0.70
Social support (CG)	MOSS (21)	Sherbourne and Stewart (1991)	0.98
Perceived stress (CG)	PSS-10 (9)	Cohen and Williamson (1988)	0.73
Anxiety (CG)	PROMIS (8)	Pilkonis et al. (2011)	0.93
Parent acceptance of violence (CG)	Individual COV (2)	Matsueda et al. (2006)	*r* = 0.35
CG inflicted inter-partner psychological aggression (CG)	CTS revised (4)	Straus et al. (1996)	0.56
CG sustained inter-partner psychological aggression (CG)	CTS revised (4)	Straus et al. (1996)	0.65
Inflicted inter-partner physical assault (CG)	CTS revised (7)	Straus et al. (1996)	0.55
Expressed inter-partner negotiation (CG)	CTS revised (3)	Straus et al. (1996)	0.69
Sustained inter-partner physical assault (CG)	CTS revised (7)	Straus et al. (1996)	0.57
Received inter-partner negotiation (CG)	CTS revised (3)	Straus et al. (1996)	0.60
Non-violent discipline for child (CG)	CTS (4)	Straus et al. (1998)	0.77
Psychological aggression (towards child) (CG)	CTS (4)	Straus et al. (1998)	0.74
Physical aggression (towards child) (CG)	CTS (3)	Straus et al. (1998)	0.62
Interpersonal			
Friend support (Y)	SSS (8)	Turner et al. (1983)	0.76
Teacher care (Y)	ADD Health (5)	Harris et al. (2019)	0.87
Trust between school and parent (Y)	TIS (5)	Bryk et al. (2002)	0.96
School belongingness (Y)	SC (6)	Resnick et al. (1997)	0.74
Perceived discrimination (Y)	EDS (9)	Forman et al. (1997)	0.87
Neighborhood			
Direct violence victimization (Y)	EtV (10)	Selner-O’Hagan et al. (1998)	–
Witness of violence victimization (Y)	EtV (11)	Selner-O’Hagan et al. (1998)	–
Neighborhood collective efficacy (CG)	PHDCN (13)	Sampson et al. (1997)	0.87
Neighborhood care (Y)	ADD Health (3)	Harris et al. (2019)	0.90
Neighborhood belongingness (CG)	ACCS (4)	Mazerolle et al. (2007)	0.88
Neighborhood social/physical disorder (CG)	PHDCN (14)	Raudenbush and Sampson (1999)	0.95
Trust in police (CG)	PHDCN (7)	Raudenbush and Sampson (1999)	0.79
Neighborhood violence (past 6 months) (CG)	PHDCN (5)	Raudenbush and Sampson (1999)	0.71

*ACCS* The Australian Community Capacity Study, *ADD Health* The National Longitudinal Study of Adolescent to Adult Health, *CES-D* Center for Epidemiological Studies Depression Scale Short Form, *COV* Code of Violence, *CTS Revised* Revised Conflict Tactics Scale, *CTS* Parent-Child Conflict Tactics Scale, *EDS* Everyday Discrimination Scale, *EtV* My Exposure to Violence Scale*,* *FES* Family Environment Scale, *LQ-SS* Loneliness Questionnaire Short Form, *MOSS* Medical Outcomes Study Social Support Survey, *MRSS* Moral Rules, Shame, and Self-control Survey, *PHDCN* Project on Human Development in Chicago Neighborhoods Survey, *PROMIS* PROMIS-Anxiety Short Form, *PSS-10* Perceived Stress Scale Short Form, *REL* Religion Scale, *SC* school connectedness, *SSS* The Social support Survey, *TIS* Trust in Schools, *Y* youth reports, *CG* caregiver reports

#### Season

The date of salivary DNA collection was used to create an indicator of season. Based on prior research indicating seasonal variation in telomere length (Kertes et al., 2022), season was dichotomized as autumn/winter (September-February) and spring/summer (March-August) for inclusion in analyses.

### Quantification of Telomere Length

Genomic DNA was isolated from 500 µL saliva via ethanol precipitation and suspended in TE buffer. Quality control was conducted via UV spectrophotometry (Nanodrop 2000, Thermo Fisher Scientific, Waltham, MA) and gel electrophoresis (1% TBE). DNA quantity and quality was verified using fluorometry (Qubit 3.0, Thermo Fisher Scientific Waltham, MA) and an automated electrophoresis system (Agilent TapeStation 2200, Santa Clara, California).

Telomere length was assessed using a commonly used metric known as relative telomere to single gene ratio (T/S ratio). Conceptually, the relative average telomere length for an individual participant is generated such that the telomere signal is normalized to the signal from a single copy gene to generate a T/S ratio. Telomere length assay protocols are reported in detail in Kertes et al. (2022). In brief, genomic DNA telomere length was assessed via quantitative polymerase chain reactions (qPCR) using telomere and single-copy gene albumin primers (Eurofins Genomics, Louisville, Kentucky) run in triplicate with HeLa genomic DNA (New England BioLabs, Ipswich, Massachusetts) to create a 5-point standard curve (1.56 to 25 ng/µL). Average reaction efficiencies were 1.87 for telomere and 1.90 for albumin. T/S ratios were computed according to standard procedures (Pfaffl, 2001). The mean R^2^ values were 0.99 for both telomere and albumin and the intraclass correlation of T/S ratios was 0.62 from a randomly selected subset of 95 samples.

### Data Analysis

Statistical analyses were conducted using R version 4.0.5. To accommodate missing item-level data, multiple imputation was conducted prior to statistical modeling using the “mice” package (van Buuren & Groothuis-Oudshoorn, 2011). Polytomous logistic regression was used for categorical data and predictive mean matching for continuous variables (Little, 1988; Venables & Ripley, 2002). All statistical models using overall measures/sum scores were carried out using Bayesian analyses, as described below. Because the statistical conclusions were drawn based on differences between the posterior (estimates after considering the data) and the prior (estimates before considering the data), using the prior allowed us to ensure that missing measure-level data had no effect on the conclusions, and an optimally minimal effect on the estimates of coefficients. This allowed us to use the prior to model a distribution of possible values for missing data at the scale level, rather than requiring imputation.

To determine the association of the predictors with the outcomes, a Bayesian regression model was implemented using JAGS [Just Another Gibbs Sampler] (Plummer, 2004; Kruschke, 2014) in MATLAB and R. Bayesian methods offer a variety of advantages over traditional regression approaches, including the ability to quantify uncertainty in terms of probability distributions over competing models (e.g., models with vs. without telomere length as a predictor of anxiety) and parameters (e.g., value of the coefficient predicting anxiety from telomere length) that cannot be obtained from classical statistical approaches. Specifically, they estimate a “posterior” distribution, I.e., a probability distribution that specifies the relative likelihoods of all possible parameter values (coefficients in the regression) given the data. The sampling procedure (JAGS) draws randomly from the posterior distribution proportional to the posterior likelihoods of different parameter values such that more likely values (e.g., those near zero, for null effects) will result in more samples near that value, while less likely values (those that reject the null) will result in fewer samples. Each chain corresponds to one sequence of samples from the posterior; when chains converge, it indicates that they all agree on the shape of the posterior. In simple terms, to test the likelihood of a particular coefficient being zero, the frequency of posterior samples near zero (indicating a null effect) relative to samples far from zero (indicating a large effect) were evaluated. Bayesian methods like these have now been used in thousands of psychology articles and have consistently been shown to generate more valid and reliable conclusions than classical regression/null hypothesis testing (van de Schoot et al., 2017). By using the posterior, these approaches quantify the likelihood of the null and various alternative hypotheses as opposed to quantifying the likelihood of the data assuming the null hypothesis is true (Wagenmakers, 2007; Wagenmakers et al., 2018), leading to both more intuitive conclusions and more flexible data analyses.

The Bayesian regression included each of the predictors outlined in Table 2 along with the interaction of each of these variables with T/S ratio were included. The effects of the set of predictors for anxiety and depressive symptoms were estimated in separate models. To estimate coefficients in the regression model and compare competing hypotheses about model structure, the posterior distribution was estimated across all parameters using a Gibbs Sampler (Plummer, 2004), with 4 chains of 5000 samples each. All chains reached convergence (r-hat statistic <1.005; Gelman & Shirley, 2011). This provided 20,000 random samples from the posterior distribution for each parameter of the model. From these samples, a 95% highest density interval [HDI] was computed specifying the 95% most likely values of each coefficient/ parameter in the model. This HDI has the benefit of being more intuitive than classical regression in which the confidence interval must strictly be interpreted as the range of values that one might expect the mean of a sample to fall within 95% of the time if an experiment were repeated many times. From the posterior samples, a Bayes factor was computed for the inclusion of each coefficient in the model by comparing the height of the prior distribution against the height of the posterior distribution at *b* = 0, referred to as a [generalized] Savage-Dickey Bayes factor (Wagenmakers et al., 2010; Heck, 2019). The estimation procedure used a standard normal distribution as the prior for each coefficient to ensure that this comparison reflected optimal inferences based on the data (Consonni et al., 2018). The Bayes factor quantifies the odds of a coefficient being nonzero, given the data available. These odds can then be transformed into a probability of inclusion “Pr(incl)” where larger values indicate that a parameter is more likely to be nonzero. Values of Pr(incl) below.5 indicate support for the null/not including a particular parameter in the model, whereas values above 0.5 indicate support for inclusion/non-null values of a parameter. Both the 95% HDI for each of the estimated parameters as well as their probability of inclusion based on the Bayes factor are reported. A credible effect in Bayesian regression is one where Pr(incl) >0.5 and HDI does not cross zero. The effects of age, sex, and season of data collection were controlled for by assigning them a prior likelihood of 1 (i.e., must be included as factors in the model). This was done to compensate for the effects of demographic and seasonal influences demonstrated in prior work in our cohort and others to covary with telomere length (Kertes et al., 2022; Ly et al., 2019).

**Table 2:** Descriptive statistics

Variable	Count/Mean	SD
Individual		
Self-control	3.302	0.744
Youth attitude to violence	2.954	0.826
Loneliness	1.819	0.859
Personal religiosity	0.312	0.836
Religious activity participation	0.086	0.900
Shame if caught by teachers	1.152	0.758
Shame if caught by parents	1.213	0.713
Shame if caught by friends	0.891	0.677
Family		
Family environment	6.805	1.737
Family support	2.601	0.409
Family care	4.655	0.661
Caregiver depression	1.743	0.518
Caregiver social support	2.944	1.081
Caregiver perceived stress	2.016	0.632
Caregiver anxiety	0.833	0.887
Parent attitude to violence	2.551	0.916
Caregiver inflicted inter-partner psychological aggression	3.377	3.951
Caregiver sustained inter-partner psychological aggression	3.323	4.422
Caregiver inflicted inter-partner physical assault	0.199	0.798
Caregiver sustained inter-partner physical assault	0.247	1.013
Caregiver expressed inter-partner negotiation	7.142	6.543
Caregiver received inter-partner negotiation	6.731	5.994
Caregiver use of non-violent discipline	5.965	5.654
Psychological aggression (towards child)	2.611	4.029
Physical aggression (towards child)	0.488	1.225
Interpersonal		
Friend support	2.594	0.364
Teacher care	4.268	0.781
Trust between school and parent	4.094	0.947
School belongingness	3.621	0.793
Experience of discrimination	2.325	0.891
Direct violence victimization	1.403	1.511
Witness of violence victimization	2.197	2.045
Neighborhood		
Neighborhood collective efficacy	3.364	0.817
Neighborhood care	2.965	1.184
Neighborhood belongingness	3.197	1.121
Neighborhood social/physical disorder	0.474	0.552
Trust in police	3.189	0.832
Neighborhood violence	0.874	0.756
Neighborhood violence	0.874	0.756

In reporting the results, common Bayesian analysis reporting guidelines (e.g., Krushke, 2014) were followed or facilitated. Sensitivity analyses for different priors can be carried out by using the priors and computations contained in the analysis code, provided at https://github.com/KertesLab/manuscript_code_repository.

## Results

### Descriptives

Of the 411 youth participants in the study (46% female), the mean age was 14.23 years (SD = 1.85). With respect to season of collection, 190 were collected in the autumn/winter and 221 in the spring/summer. Mean T/S ratio was 0.29 (SD = 0.81). In this community-based sample of adolescents, anxiety symptoms (*M* = 1.76, SD = 0.71) and depressive symptoms (*M* = 0.82, SD = 0.55) was similar to other populations unselected for clinical services or diagnosis (Ialongo et al., 2004). Descriptive statistics for the array of personal, family, interpersonal, and neighborhood level social moderators are shown in Table 2. A correlation matrix is provided in Supplementary Information.

### Best-Fitting Predictors with Direct Association to Anxiety Symptoms

**Fig. 1:**
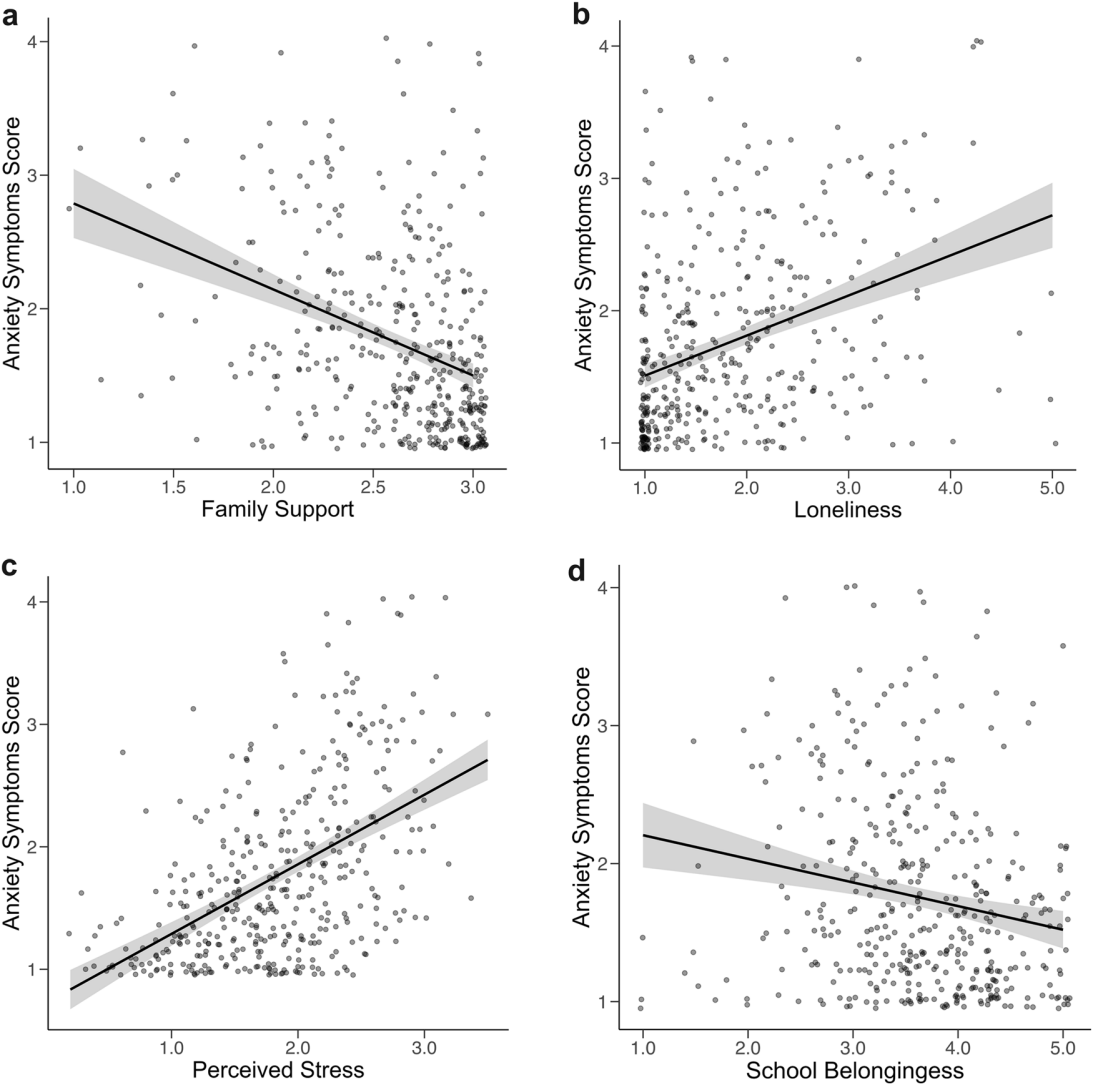
**a**–**d** Direct association of social environment with anxiety symptoms. **a** Family support, **b** Loneliness, **c** Perceived stress, **d** School belongingness

**Fig. 2:**
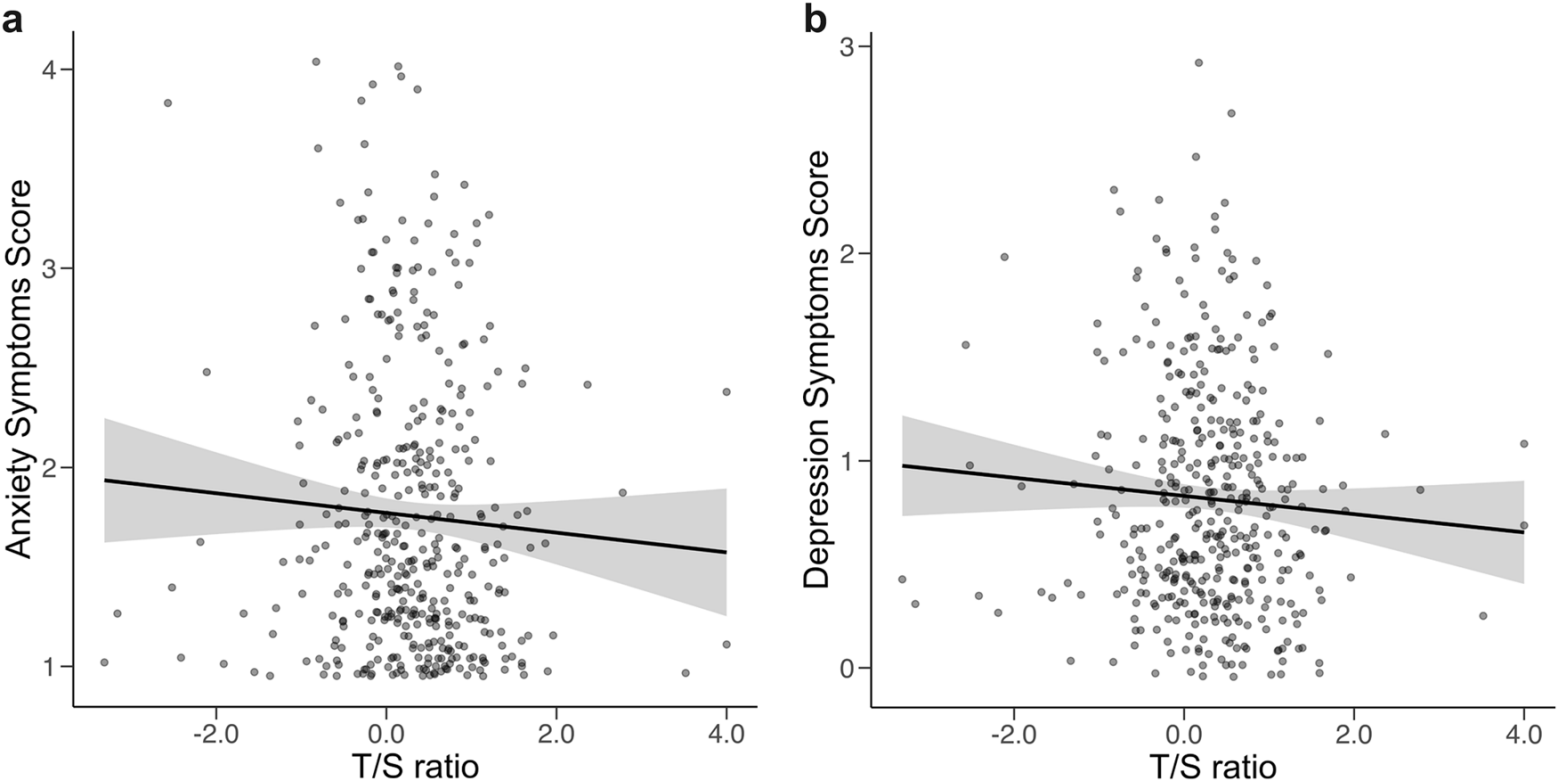
Direct association of T/S ratio with anxiety and depressive symptoms. **a** Anxiety symptoms, **b** Depressive symptoms

The results showed that family support (*M* = −0.14, 95% HDI = [−0.21, −0.05], BF = 3.17, Pr(incl|data) = 0.76), loneliness (*M* = 0.14, 95% HDI = [0.04, 0.23], BF = 2.22, Pr(incl|data) = 0.69), perceived stress (*M* = 0.28, 95% HDI = [0.21, 0.37], BF = 36.24, Pr(incl|data) = 0.97), and school belongingness (*M* = 0.10, 95% HDI = [0.03, 0.19], BF = 1.04, Pr(incl|data) = 0.51) were significantly associated with anxiety symptoms. Specifically, lower family support, lower school belongingness, higher perceived stress, and higher loneliness were associated with higher levels of youth anxiety symptoms. Figure 1a–d shows the credible direct social environment effects on anxiety symptoms. Figure 2a displays T/S ratio with anxiety symptoms, where modest trends for smaller T/S ratio (indicating shorter telomere length) and higher anxiety symptoms are observed; however, this direct association were not identified in the best fitting Bayes model.

### Social Environment Moderates the Association of Telomere Length and Anxiety Symptoms

**Fig. 3:**
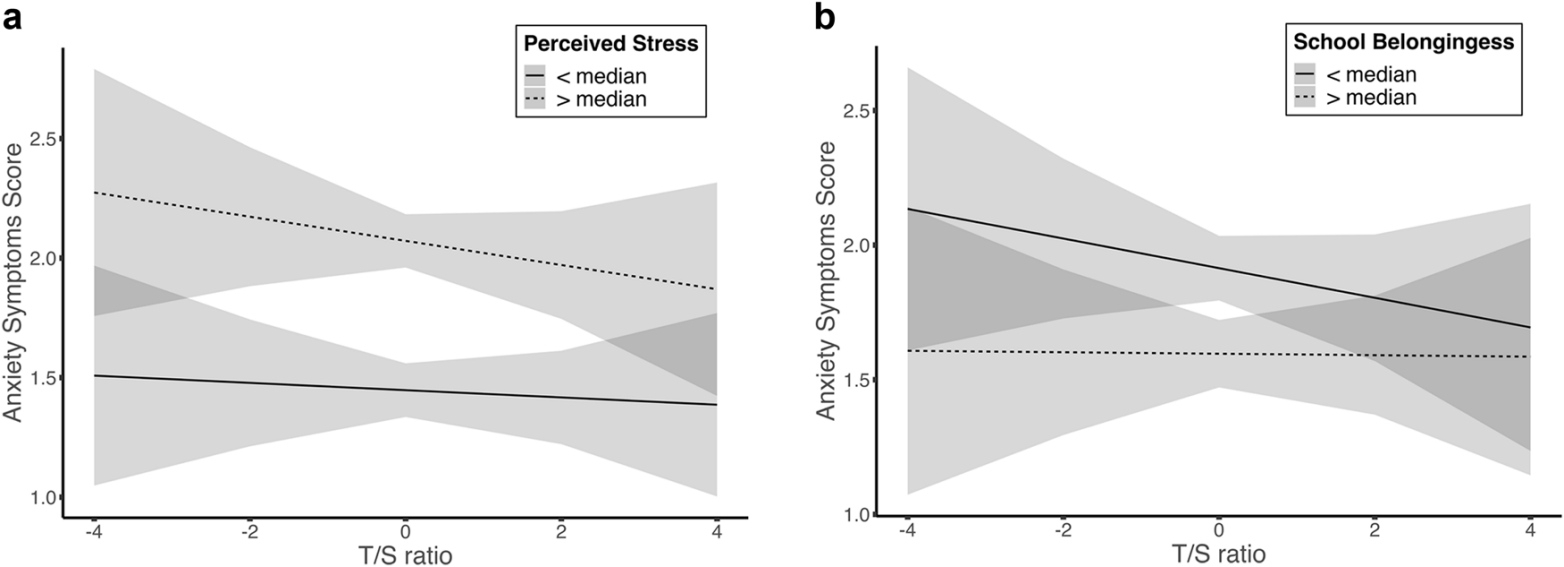
Moderation effect of **a** perceived stress and **b** school belongingess on telomere length - anxiety symptom associations

There was a credible interaction between T/S ratio and perceived stress on youth anxiety symptoms (*M* = −0.15, 95% HDI = [−0.26, −0.03], BF = 1.33, Pr(incl|data) = 0.57), indicating that adolescents with larger T/S ratios (reflecting longer telomere length) and lower perceived stress had lower anxiety symptoms, over and above the additive effects of telomere length or perceived stress alone. An illustration of this moderated effect is shown in Fig. 3a, with a median split on levels of perceived stress plotted as dashed (above the median) and solid lines (below the median). As shown in the figure, the effect of longer telomere length related to lower anxiety symptoms was more pronounced in children under higher levels of perceived stress, which was comparatively mild among children with lower levels of perceived stress.

There was also an interaction between T/S ratio and school belongingness that was credible on anxiety symptoms (*M* = −0.16, 95% HDI = [−0.27, −0.04], BF = 1.82, Pr(incl|data) = 0.65) indicating that adolescents with larger T/S ratio (reflecting longer telomere length) and higher school belongingness had lower anxiety, over and above the additive effects of telomere length or school belongingness separately. As illustrated in Fig. 3b, the effect of longer telomere length related to lower anxiety symptoms was more pronounced in children under lower levels of school belongingness, which was comparatively mild among children with higher levels of school belongingness.

### Best-Fitting Predictors with Direct Associations to Depressive Symptoms

**Fig. 4:**
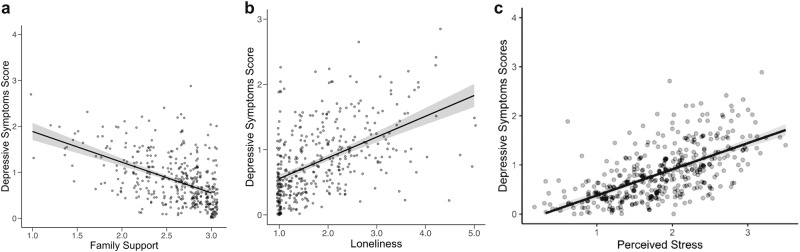
**a**–**c** Direct association of social environment with depressive symptoms. **a** Family support, **b** Loneliness, **c** Perceived stress

Paralleling the null association between T/S ratio and anxiety symptoms, modest trends for smaller T/S ratios (indicating shorter telomere length) and higher depressive symptoms were observed (see Fig. 2b), although these associations were not identified in the best fitting model. Significant direct effects of family support (*M* = −0.16, 95% HDI = [−0.21, −0.10], BF = 25.13, Pr(incl|data) = 0.96), perceived stress (*M* = 0.23, 95% HDI = [0.18, 0.28], BF = 23.33, Pr(incl|data) = 0.96), and loneliness (*M* = 0.12, 95% HDI = [0.06, 0.18], BF = 15.55, Pr(incl|data) = 0.94) were also observed. Similar to findings involving the anxiety symptoms as the outcome, lower family support, higher perceived stress, and higher loneliness were associated with higher depressive symptoms (see Fig. 4a–c).

### Social Environment Moderates the Association of Telomere Length and Depressive Symptoms

**Fig. 5:**
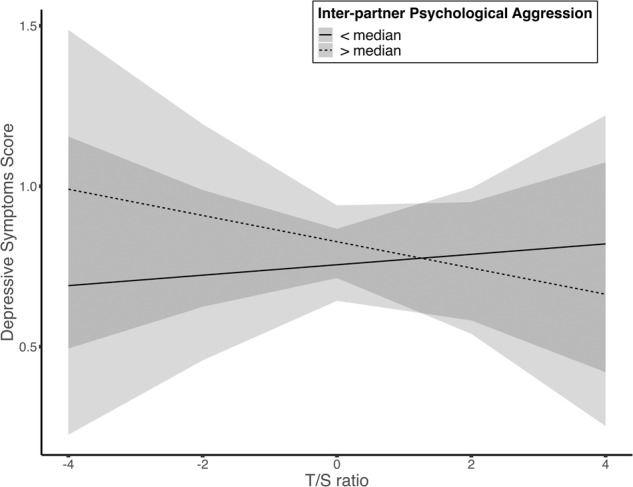
Moderation effect of caregiver inter-partner psychological aggression on telomere length –depressive symptom associations

There was a credible interaction of T/S ratio and parent inter-partner psychological aggression on depressive symptoms (*M* = −0.14, 95% HDI = [−0.23, −0.04], BF = 2.02, Pr(incl|data) = 0.67) such that adolescents with larger T/S ratio and lower levels of parent inter-partner aggression had lower depressive symptoms, above and beyond the additive effects of either predictor alone. The moderated effect of inter-partner psychological aggression is shown in Fig. 5. Similar to the previous visualization process, a median split of caregiver use of inter-partner psychological aggression was performed and plotted the relation between T/S ratio and depressive symptoms. As shown in the figure, for adolescents living with higher levels of parent inter-partner psychological aggression, longer telomere length was related to lower levels of depressive symptoms, whereas the effect was not evident among adolescents living with lower levels of parent inter-partner psychological aggression. Stated differently, adolescents with short T/S ratios appear to be more prone to depressive symptoms when in the presence of a high degree of parent inter-partner psychological aggression.

## Discussion

Despite strong evidence that internalizing symptoms that manifest during adolescence have long-term implications for mental and physical health across the lifespan, little is known about the individual specific and contextual risk factors that impact risk for internalizing problems, particularly among Black adolescents who show rising rates and carry a disproportionate burden of internalizing symptoms. Addressing this gap is critical to reduce health disparities and inform an understanding of environmental exposures that influence the epigenetic landscape and internalizing psychopathology. Leveraging a novel Bayesian statistical method, joint contributions of potentially malleable factors, including indices of biological aging (i.e., telomere length) and the social environment, were examined with internalizing symptoms during adolescence, a crucial developmental window characterized by increased susceptibility to contextual factors.

The results of this study revealed several novel interactive effects linking telomere length, the social environment, and anxiety and depressive symptoms in adolescence. In the best fitting (Bayesian) regression model, the results demonstrated that the association of TL and anxiety symptoms was moderated by perceived stress, such that the telomere length—anxiety symptom association was more pronounced among youth with higher perceived stress. In other words, telomere length and anxiety symptoms were more tightly coupled under conditions of higher perceived stress. Although adolescence is marked by normative increases in perceived stress (Spear, 2009), it is possible that adolescents who report higher levels of perceived stress may be exposed to more frequent and severe contextual stressors (Heinze et al., 2017), low self-esteem or self-efficacy (Piekarska, 2020), or may experience higher levels of negative affective and behavioral states (Felton et al., 2017; Cook et al., 2012) that make them more likely to interpret situations as stressful. It is also possible that cognitive appraisals of situations as more stressful may activate stress-related neurobiological systems including the hypothalamic-pituitary adrenal axis (HPA), autonomic nervous system (ANS), and promote inflammation (O’Donovan et al., 2013). Higher perceived stress may contribute to dysregulation of neural stress response systems and changes in immune cell receptor functioning, both of which may contribute to telomere shortening and increased risk for anxiety symptoms (O’Donovan et al., 2013), leading to a stronger coupling of these two markers of health.

School belongingness also moderated the association of telomere length with anxiety symptoms such that greater school belongingness attenuated the association between shorter telomere length and anxiety symptoms. In line with social ecological frameworks and the integrative model of development (Bronfenbrenner, 1977; Garcia Coll et al., 1996), promotive school environments may serve as sources of resilience for adolescents by providing them with the opportunity to form supportive social networks with peers and teachers alike, which may increase positive affect, self-efficacy, and self-esteem. Similarly, youth who report greater school affiliation may be more likely to employ healthy coping strategies and may be more adept at self-regulating negative emotions in response to stressors (Arslan, 2021). Concurrently, shorter telomere length may reflect the accumulation of prior risk from the lens of the diathesis-stress model, such that shorter telomere length under conditions of low school belongingness as a social stressor is linked with higher anxiety symptoms. In this study, shorter telomere length and higher anxiety symptoms was more strongly coupled under conditions of low school belongingness than higher school belongingness. Given that feelings of belonginess have been previously identified as a positive social determinant of mental (Santamaría-García et al., 2020) and physical health outcomes (Michalski et al., 2020) across the life span, the present results provide suggestive evidence for the potential benefit of school connection in decoupling cellular aging and anxiety symptoms.

A significant interaction was also observed for parent inter-partner psychological aggression and telomere length with depressive symptoms. Inter-partner psychological aggression reflected the degree to which caregivers in the household yell, shout, insult, swear, destroy belongings, or threaten to hit their partner. In the context of greater parent inter-partner psychological aggression, shorter telomere length was associated with higher depressive symptoms. Although numerous studies have documented the effects of witnessing inter-partner psychological aggression during the early childhood years (Artz et al., 2014; Caldeira & Woodin, 2012), exposure to these behaviors may also have impacts during adolescence, a developmental period characterized by increased vulnerability to context and rapid changes in brain maturation (Schiff et al., 2014), along with a developmentally different understanding of inter-partner relationships than children. This study did not assess the history of inter-partner aggression prior to study enrollment. Thus, it is not possible to fully disentangle whether the effect of inter-partner aggression is due to longer-term or recent exposure to this type of interaction in adolescence only. In either case, it is plausible that adolescents with exposure to the chronic stress of inter-partner psychological aggression may evidence prolonged activation of physiological response systems (even when the stressor has dissipated) (Howell et al., 2016) which may be associated with telomere length erosion and exacerbate risk for depression symptoms (Jiang et al., 2019).

In addition to the novel interaction effects that were the focus of the study, several main effects of the social environment on anxiety and depressive symptoms were observed in this study. Lower family support, higher perceived stress, and higher loneliness were associated with higher levels of youth anxiety and depressive symptoms, whereas lower school belongingness was uniquely associated with greater anxiety symptoms. Prior research using GLM based models have linked lower family support (Guberman & Manassis, 2011), higher levels of loneliness (Danneel et al., 2019; Ebesutani et al., 2015), and perceived stress (Felton et al., 2017) to heightened risk for internalizing symptoms, and greater school connection to school during the high school years with lower internalizing symptoms (Arslan, 2021; Pittman & Richmond, 2007). The present findings are in line with both the risk and resilience model of developmental psychopathology (Cicchetti & Rogosch, 2002; Andreotti et al., 2013) and integrative model of development (Garcia Coll et al., 1996) as they highlight the association of environmental stressors with psychopathological symptoms during racial minority youth development. Taken together, the present findings using a Bayesian modeling approach highlight that support within the family system, school belongingness, perceived stress, and loneliness are among the most robust individual-specific and environmental correlates that are associated with the manifestation of internalizing problems among Black adolescents, enhancing an understanding of factors linked to anxiety and depressive symptoms in this population.

The results of this study should be interpreted in light of the population, study design, and analytic strategy, which affords both strengths and limitations. First, the study population were Black adolescent youth in the U.S., which may limit generalizability to other demographic groups. The focus on a historically marginalized population, both in terms of social and economic opportunity as well as biobehavioral research, was a deliberate choice to address the broader goal of improving the understanding of minority health. No a priori assumptions were made that findings from prior studies conducted in primarily White or mixed-race adolescents inherently generalize to Black youth; in contrast, this study adopted a minority youth development approach that centers on the experiences of marginalized youth.

Second, this study utilized Bayesian modeling approach to identify the strongest set of predictors of a target outcome, estimate predictor strength, and assign each a probability that quantifies how important it is to include in the regression model. Whereas Bayesian model results are not directly comparable to “significance” testing conducted under GLM-based models, the Bayesian approach had notable advantages: first, it enabled us to reject the inclusion of certain variables (accept the null); second, it permitted us to include important demographic and seasonal differences across participants by default (setting priors to 1); and third, it allowed us to identify the strongest set of predictors given the totality of data in a complex, real-world dataset, allowing it to more cleanly estimate the credible effects in absence of non-credible effects.

Finally, a limitation of the present study is the cross-sectional study design. Longitudinal methods would be needed to clearly parse apart directionality between mental health, biological aging, and social context. In all likelihood, bi- or multi-directional effects may occur, whereby the emergence of internalizing symptoms may put additional strain on both social relations and health behaviors that impact stress-sensitive neurobiological systems, inflammatory responses and oxidative stress, further eroding telomere integrity. At present, interpretation of directionality effects even in longitudinal samples is limited by technological challenges to accurately comparing telomere length for samples collected over the span of several years (Nettle et al., 2021). Nevertheless, the present findings conducted with a population of over 400 Black youth primarily from low- to middle- income neighborhoods offer an important step forward towards understanding biological aging in a population that is historically marginalized and under-represented in research, among whom biological aging occurs more rapidly than their White counterparts, and who are disproportionately burdened by mental and physical health disorders.

## Conclusion

With depression and anxiety symptoms are on the rise among adolescents and growing evidence that cellular aging may be associated with depressive and anxiety symptoms, there is an urgent need to identify the social environment context that modulates this link. This study addressed this research gap by investigating the moderating role of the social environment on the relation between telomere length and emotional health in youth. Using a Bayesian modeling approach to identify the most robust interaction effects, the results of the study demonstrated that the social environment moderated the association of telomere length with internalizing problems among Black youth. Specifically, the association of shorter telomere length and higher anxiety symptoms was stronger under conditions of low school belongingness than higher school belongingness, and high perceived stress compared to lower perceived stress. In addition, adolescents with shorter telomeres were more likely to exhibit depressive symptoms under conditions of living with high parent inter-partner psychological aggression. Stated differently, adolescents with shorter telomeres were more prone to depressive symptoms if they had parents with high inter-partner aggression, and more prone to anxiety symptoms if they had higher perceived stress or low school belongingness. Notably, among children with higher levels of school belongingness or lower perceived stress, associations between telomere length and anxiety symptoms were comparatively mild. There are several implications of these findings. First, from a preventive intervention lens, perceived stress and school belongingness may be potentially modifiable targets for preventive intervention to reduce the burden of anxiety among Black youth, and that intervention efforts focused on depressive symptoms among Black youth may need to carefully consider adverse experiences within the home when tailoring intervention efforts. Second, from an empirical and conceptual lens, the results speak to the need to model social context in understanding the relation of biological aging and mental health and more broadly, the interaction of biological factors such as telomere length that reflect the accumulation of early life experiences with the present social environment. Third, from a methodological lens, given the “distance” between the social environment and chromosomes—including cognitive, emotional, and physiological systems, observed association between variation in the social environment and telomere length is expected to be small and difficult to detect statistically. Associations detected with a robust analytic strategy such as Bayesian modeling—which affords the ability to assign confidence to credible effects—are noteworthy and indicate strong confidence that social context moderates the association of biological aging and internalizing problems. Overall, this study adds novel information to the literature on telomere length with mental health by highlighting ways in which the social environment matters for understanding the link between biological aging and mental health.

### Supplementary Information

Supplementary Information
