# Supplementary material for: The Social Environment Matters for Telomere Length and Internalizing Problems During Adolescence
Source: J Youth Adolesc. 2023 Sep 25;53(1):21–35. doi: 10.1007/s10964-023-01848-w (PMC10761382; doi:10.1007/s10964-023-01848-w)
Supplement: Supplementary file 1 — Supplementary Information [file 10964_2023_1848_MOESM1_ESM.docx]

**Supplementary Information**

**Supplementary Table 1. Correlations among Social Environment Predictors**

|  | 1 | 2 | 3 | 4 | 5 | 6 | 7 | 8 | 9 | 10 | 11 | 12 | 13 | 14 | 15 | 16 | 17 | 18 | 19 | 20 | 21 | 22 | 23 | 24 | 25 | 26 | 27 | 28 | 29 | 30 | 31 | 32 | 33 | 34 | 35 | 36 | 37 | 38 | 39 | 40 | 41 | 42 | 43 | 44 |
| --- | --- | --- | --- | --- | --- | --- | --- | --- | --- | --- | --- | --- | --- | --- | --- | --- | --- | --- | --- | --- | --- | --- | --- | --- | --- | --- | --- | --- | --- | --- | --- | --- | --- | --- | --- | --- | --- | --- | --- | --- | --- | --- | --- | --- |
| (1) Age | **-** |  |  |  |  |  |  |  |  |  |  |  |  |  |  |  |  |  |  |  |  |  |  |  |  |  |  |  |  |  |  |  |  |  |  |  |  |  |  |  |  |  |  |  |
| (2) Season | **.19** | - |  |  |  |  |  |  |  |  |  |  |  |  |  |  |  |  |  |  |  |  |  |  |  |  |  |  |  |  |  |  |  |  |  |  |  |  |  |  |  |  |  |  |
| (3) Anxiety | **.11** | **.07** | - |  |  |  |  |  |  |  |  |  |  |  |  |  |  |  |  |  |  |  |  |  |  |  |  |  |  |  |  |  |  |  |  |  |  |  |  |  |  |  |  |  |
| (4) Depression | **.07** | **.02** | **.62** | - |  |  |  |  |  |  |  |  |  |  |  |  |  |  |  |  |  |  |  |  |  |  |  |  |  |  |  |  |  |  |  |  |  |  |  |  |  |  |  |  |
| (5) T/S Ratio | -.11 | -.17 | -.04 | -.01 | - |  |  |  |  |  |  |  |  |  |  |  |  |  |  |  |  |  |  |  |  |  |  |  |  |  |  |  |  |  |  |  |  |  |  |  |  |  |  |  |
| (6) CG anxiety | **-.09** | -.08 | **.09** | **.11** | -.06 | - |  |  |  |  |  |  |  |  |  |  |  |  |  |  |  |  |  |  |  |  |  |  |  |  |  |  |  |  |  |  |  |  |  |  |  |  |  |  |
| (7) CG depression | .01 | -.07 | **.17** | **.16** | -.05 | **.51** | - |  |  |  |  |  |  |  |  |  |  |  |  |  |  |  |  |  |  |  |  |  |  |  |  |  |  |  |  |  |  |  |  |  |  |  |  |  |
| (8) CG EI negotiation | .03 | **-.05** | .05 | .03 | .04 | .03 | .03 | - |  |  |  |  |  |  |  |  |  |  |  |  |  |  |  |  |  |  |  |  |  |  |  |  |  |  |  |  |  |  |  |  |  |  |  |  |
| (9) CG II physical assault | .03 | **-.06** | **.06** | **.06** | .04 | **.10** | **.24** | .17 | - |  |  |  |  |  |  |  |  |  |  |  |  |  |  |  |  |  |  |  |  |  |  |  |  |  |  |  |  |  |  |  |  |  |  |  |
| (10) CG perceived stress | .00 | **-.07** | **.10** | **.10** | -.09 | **.56** | **.50** | .16 | **.05** | - |  |  |  |  |  |  |  |  |  |  |  |  |  |  |  |  |  |  |  |  |  |  |  |  |  |  |  |  |  |  |  |  |  |  |
| (11) CG RI negotiation | -.04 | **-.09** | -.02 | .00 | **.10** | .00 | -.04 | **.74** | .15 | .05 | - |  |  |  |  |  |  |  |  |  |  |  |  |  |  |  |  |  |  |  |  |  |  |  |  |  |  |  |  |  |  |  |  |  |
| (12) CG social support | -.02 | **.12** | .04 | **.00** | .04 | **-.26** | **-.37** | .02 | **-.16** | **-.34** | .06 | - |  |  |  |  |  |  |  |  |  |  |  |  |  |  |  |  |  |  |  |  |  |  |  |  |  |  |  |  |  |  |  |  |
| (13) CG SI physical assault | -.04 | **-.01** | .04 | **.03** | .02 | **.06** | **.22** | .23 | **.59** | **.08** | .18 | **-.14** | - |  |  |  |  |  |  |  |  |  |  |  |  |  |  |  |  |  |  |  |  |  |  |  |  |  |  |  |  |  |  |  |
| (14) CG use of non-violent discipline | **-.26** | **-.04** | .05 | .06 | -.06 | **.12** | **.08** | **.27** | **-.02** | **.13** | .21 | **-.09** | **.12** | - |  |  |  |  |  |  |  |  |  |  |  |  |  |  |  |  |  |  |  |  |  |  |  |  |  |  |  |  |  |  |
| (15) CG II psych aggression | .00 | **-.09** | .06 | .04 | .07 | **.17** | **.21** | **.55** | **.41** | **.22** | **.49** | **-.12** | **.41** | **.09** | - |  |  |  |  |  |  |  |  |  |  |  |  |  |  |  |  |  |  |  |  |  |  |  |  |  |  |  |  |  |
| (16) CG SI psych aggression | .00 | **.01** | **.05** | **.06** | .05 | **.13** | **.22** | **.59** | **.41** | **.23** | **.50** | **-.17** | **.42** | **.14** | **.79** | - |  |  |  |  |  |  |  |  |  |  |  |  |  |  |  |  |  |  |  |  |  |  |  |  |  |  |  |  |
| (17) Collective efficacy | .03 | .10 | **.02** | .07 | **.06** | **-.16** | **-.17** | .09 | **-.14** | **-.08** | .09 | **.24** | **-.16** | **.05** | **-.02** | **.02** | - |  |  |  |  |  |  |  |  |  |  |  |  |  |  |  |  |  |  |  |  |  |  |  |  |  |  |  |
| (18) Direct violence victimization | **.12** | **.05** | **.29** | **.28** | **-.04** | -.01 | .01 | .03 | -.11 | -.02 | **-.06** | .03 | .01 | .09 | -.09 | -.08 | .02 | - |  |  |  |  |  |  |  |  |  |  |  |  |  |  |  |  |  |  |  |  |  |  |  |  |  |  |
| (19) Family care | **-.16** | **-.02** | **-.21** | **-.28** | **-.04** | -.10 | **-.07** | .06 | .04 | -.04 | **.05** | .05 | .08 | .06 | .11 | .07 | .02 | **-.22** | - |  |  |  |  |  |  |  |  |  |  |  |  |  |  |  |  |  |  |  |  |  |  |  |  |  |
| (20) Family environment | -.10 | -.01 | **-.13** | **-.12** | .01 | **-.09** | **-.27** | -.10 | **-.18** | **-.19** | -.04 | **.21** | **-.10** | **-.06** | **-.09** | **-.17** | **.22** | **-.06** | **.07** | - |  |  |  |  |  |  |  |  |  |  |  |  |  |  |  |  |  |  |  |  |  |  |  |  |
| (21) Family support | **-.21** | -.03 | **-.41** | **-.51** | -.04 | **-.09** | **-.21** | .01 | **-.10** | **-.08** | .02 | **.11** | **-.09** | .09 | **-.04** | **-.08** | **.10** | **-.26** | **.50** | **.21** | - |  |  |  |  |  |  |  |  |  |  |  |  |  |  |  |  |  |  |  |  |  |  |  |
| (22) Friend support | .02 | .01 | **-.23** | **-.35** | **.07** | **-.12** | **-.20** | .04 | **-.10** | **-.09** | **.08** | **.06** | **-.06** | -.02 | .04 | **-.02** | **.11** | **-.13** | **.30** | **.13** | **.32** | - |  |  |  |  |  |  |  |  |  |  |  |  |  |  |  |  |  |  |  |  |  |  |
| (23) Loneliness | **.12** | **.09** | **.42** | **.51** | .02 | **.09** | **.19** | -.01 | **.09** | **.13** | **-.03** | **-.03** | **.05** | -.03 | .06 | **.08** | **-.01** | **.21** | **-.36** | **-.09** | **-.46** | **-.47** | - |  |  |  |  |  |  |  |  |  |  |  |  |  |  |  |  |  |  |  |  |  |
| (24) NBRHD Belongingness | **.13** | **.10** | .06 | .05 | .11 | **-.17** | **-.17** | .01 | **-.17** | **-.15** | .02 | **.23** | **-.13** | **-.05** | **-.08** | **-.01** | **.62** | .08 | -.09 | **.17** | .01 | **.13** | .03 | - |  |  |  |  |  |  |  |  |  |  |  |  |  |  |  |  |  |  |  |  |
| (25) NBRHD Care | **-.26** | .01 | **-.10** | **-.05** | .01 | .07 | .10 | -.07 | -.02 | .05 | -.06 | -.02 | .04 | .18 | -.12 | -.04 | .24 | **-.10** | **.12** | .05 | **.12** | **.02** | **-.09** | .15 | - |  |  |  |  |  |  |  |  |  |  |  |  |  |  |  |  |  |  |  |
| (26) NBRHD Social/Physical disorder | -.14 | **-.11** | **-.01** | **.04** | -.07 | **.15** | **.29** | -.01 | **.25** | **.10** | -.02 | **-.28** | **.20** | **.11** | **.12** | **.09** | **-.53** | -.01 | -.06 | **-.19** | **-.14** | **-.08** | **.01** | **-.48** | -.01 | - |  |  |  |  |  |  |  |  |  |  |  |  |  |  |  |  |  |  |
| (27) NBRHD Violence | **-.10** | **-.10** | .01 | -.04 | -.06 | **.17** | **.14** | .01 | **.06** | **.06** | -.04 | **-.11** | **.09** | **.08** | **.02** | **-.01** | **-.40** | .06 | .01 | **-.02** | -.01 | **-.07** | -.01 | **-.32** | -.09 | **.48** | - |  |  |  |  |  |  |  |  |  |  |  |  |  |  |  |  |  |
| (28) Parent acceptance of violence | .04 | .09 | -.12 | -.02 | -.04 | **.01** | **.05** | -.05 | **.13** | **.06** | -.09 | **-.03** | **.10** | -.04 | **.06** | **.07** | **-.10** | -.02 | .07 | **-.06** | **.03** | **-.06** | **.08** | **-.12** | -.05 | **.10** | **.08** | - |  |  |  |  |  |  |  |  |  |  |  |  |  |  |  |  |
| (29) Perceived discrimination | **.12** | **.15** | **.39** | **.48** | -.02 | **.03** | **.13** | -.02 | .01 | **-.01** | **-.04** | -.02 | .01 | .06 | -.04 | .00 | .05 | **.41** | **-.32** | **-.09** | **-.42** | **-.38** | **.50** | .03 | **-.08** | .04 | .01 | -.04 | - |  |  |  |  |  |  |  |  |  |  |  |  |  |  |  |
| (30) Personal religiosity | **-.11** | .04 | .03 | **-.05** | -.02 | .03 | .01 | -.01 | -.04 | .04 | .03 | .00 | .04 | **.11** | -.05 | -.04 | -.06 | .01 | **.07** | -.01 | **.23** | .07 | **-.08** | **-.09** | **.11** | .04 | .05 | .00 | **-.11** | - |  |  |  |  |  |  |  |  |  |  |  |  |  |  |
| (31) Physical aggression TC | **-.27** | **-.05** | .01 | -.06 | .13 | **.12** | **-.02** | .00 | **.03** | **.10** | -.02 | **-.13** | **.05** | **.36** | **.05** | **.05** | **-.04** | .00 | .11 | **-.10** | .13 | -.02 | .01 | **-.09** | **.05** | **.05** | **.12** | **.04** | -.04 | **.17** | - |  |  |  |  |  |  |  |  |  |  |  |  |  |
| (32) Psych aggression TC | -.15 | **-.10** | **.09** | **.11** | .07 | **.16** | **.11** | **.11** | **.08** | **.28** | .12 | **-.23** | **.14** | **.43** | **.19** | **.17** | **-.14** | .08 | .06 | **-.14** | **-.02** | **-.11** | **.10** | **-.17** | -.01 | **.12** | **.12** | **.10** | .06 | .09 | **.49** | - |  |  |  |  |  |  |  |  |  |  |  |  |
| (33) Religiosity | **-.11** | .04 | .07 | **.00** | .02 | .04 | -.02 | .04 | **-.07** | -.01 | .06 | .00 | .01 | **.16** | -.09 | **-.05** | -.04 | .05 | **.07** | -.03 | **.17** | .03 | **-.07** | -.05 | **.14** | -.02 | -.04 | -.03 | .00 | **.73** | **.15** | .04 | - |  |  |  |  |  |  |  |  |  |  |  |
| (34) Religious activity participation | -.05 | .05 | .06 | **.00** | .00 | **-.03** | **-.07** | .04 | **-.11** | **-.07** | .02 | **.07** | **-.07** | .17 | **-.12** | **-.12** | **.03** | .06 | **.07** | **.04** | **.12** | **-.03** | **-.05** | **.03** | .03 | **-.10** | **-.07** | **-.06** | .03 | **.37** | .12 | **-.03** | **.72** | - |  |  |  |  |  |  |  |  |  |  |
| (35) School belongingness | **-.22** | **-.10** | **-.23** | **-.38** | .11 | **.02** | **-.06** | .06 | **.00** | **-.05** | **.04** | -.02 | -.02 | .07 | .01 | .00 | **.06** | **-.21** | **.26** | **.15** | **.35** | **.34** | **-.43** | .04 | **.27** | .02 | .05 | **-.05** | **-.37** | **.04** | .04 | **-.06** | **.08** | .06 | - |  |  |  |  |  |  |  |  |  |
| (36) Self Control | **-.01** | -.09 | **-.31** | **-.43** | -.05 | **-.01** | **-.08** | -.07 | **-.07** | **-.04** | .01 | -.06 | **.00** | -.09 | -.04 | **-.12** | -.05 | **-.19** | **.12** | **.21** | **.36** | **.28** | **-.31** | -.01 | **-.02** | **-.04** | -.03 | **-.03** | **-.34** | **.13** | .00 | **-.08** | **.05** | **.02** | **.21** | - |  |  |  |  |  |  |  |  |
| (37) Shame if caught by friends | .04 | -.05 | **-.08** | **-.18** | .05 | **-.09** | **-.19** | .08 | **-.20** | **.00** | **.04** | **.13** | **-.13** | **-.05** | **-.06** | **-.10** | **.14** | **-.05** | **.13** | **.29** | **.26** | **.19** | **-.13** | **.12** | -.07 | **-.25** | **-.10** | **-.09** | **-.18** | .06 | -.07 | **.01** | **.06** | **.10** | **.03** | **.27** | - |  |  |  |  |  |  |  |
| (38) Shame if caught by parents | .14 | .05 | **-.07** | **-.11** | -.09 | **-.03** | **-.10** | .11 | **-.09** | **.00** | .06 | **.08** | **-.08** | -.05 | **.00** | **-.04** | **.04** | .04 | **.10** | **.19** | **.15** | **.06** | **-.05** | **.12** | -.09 | **-.19** | **-.11** | **-.04** | **-.09** | -.03 | **-.07** | **-.01** | .04 | **.13** | -.07 | **.23** | **.59** | - |  |  |  |  |  |  |
| (39) Shame if caught by teachers | .13 | -.04 | **-.14** | **-.20** | -.01 | **-.06** | **-.14** | .09 | **-.11** | **-.06** | .04 | **.02** | **-.04** | **-.07** | **-.03** | **-.06** | **.03** | **.01** | **.11** | **.19** | **.16** | **.13** | **-.14** | **.05** | -.02 | **-.14** | **-.02** | **.00** | **-.15** | .01 | -.05 | **-.01** | .05 | **.10** | **.09** | **.27** | **.69** | **.63** | - |  |  |  |  |  |
| (40) Teacher care | **-.14** | -.02 | **-.11** | **-.20** | **.06** | -.04 | .03 | **.11** | .05 | -.04 | **.13** | -.05 | .13 | .08 | .10 | .08 | .06 | **-.12** | **.39** | -.04 | **.21** | **.34** | **-.27** | .04 | **.23** | .07 | .04 | .04 | **-.22** | **.10** | **.01** | .02 | **.09** | .02 | **.47** | **.12** | **.03** | -.05 | **.11** | - |  |  |  |  |
| (41) Trust between school and parent | -.03 | -.09 | **-.13** | **-.16** | **.06** | **-.10** | **-.08** | .08 | **-.05** | **-.08** | **.10** | **.13** | **-.11** | **-.10** | .03 | .00 | **.11** | **-.10** | **.05** | **.10** | **.06** | **.16** | **-.11** | **.10** | -.05 | **-.06** | **-.03** | **-.19** | **-.18** | -.10 | -.04 | **-.17** | -.10 | -.07 | **.21** | **.03** | **.08** | **.00** | .07 | **.16** | - |  |  |  |
| (42) Trust in police -.03 .02 | | | **-.01 .02** | | **.07** | **-.11** | **-.12** | .00 **-.06** | | **-.14** | -.01 | **.08** | **.01** | **-.06** | **.00 -.03** | | **.33** | **-.10 -.01** | | **.16** | **.05** | **.01** | **.01** | **.26** | **.18** | **-.27** | **-.35 -.10** | | **-.06** -.07 -.01 | | | **-.10** -.01 | | .03 **.08 .07 .02 -.03** | | | | | .00 | .02 **.03** - | | |  |  |
| (43) Witness of violence victimization **.18 .05** | | | **.13 .06** | | **-.08** | -.10 | .04 | .01 -.05 | | -.07 | **-.04** | .01 | .06 | .02 | -.04 -.01 | | -.01 | **.56 -.11** | | **-.03** | **-.14** | **-.07** | **.13** | .02 | **-.08** | -.02 | .04 .05 | | **.29** .04 -.08 | | | .02 .01 | | .03 **-.14 -.05 -.10** .03 | | | | | .00 | **-.09 -.10 -.15** | | | - |  |
| (44) Youth attitude of violence **.02 .19** | | | **.21 .24** | | **-.05** | **-.03** | **.07** | -.03 **.13** | | **.00** | **-.07** | **.02** | **.13** | -.03 | .01 **.08** | | **-.06** | **.32 -.11** | | **-.17** | **-.21** | **-.17** | **.24** | **-.04** | **-.04** | **.05** | **.03 .18** | | **.35** .05 -.02 | | | **.01 -.02** | | **-.05 -.21 -.48 -.29 -.27** | | | | | -.27 | **-.13 -.15 -.08** | | | **.22** | - |

Note: Bold indicates p<0.05; CG=Care giver, EI= expressed inter-partner, II= inflicted inter-partner, NBRHD= Neighborhood, psych= psychological, RI= received inter-partner, SI= sustained inter-partner, TC= towards child
